# A Patient-Centered Ethical Framework for Irritable Bowel Syndrome Care: Communication, Trust, Nutrition-Sensitive Care, and Self-Management

**DOI:** 10.3390/nu18132036

**Published:** 2026-06-23

**Authors:** Ioanna Aggeletopoulou, Ploutarchos Pastras, Alexandra K. Tsaroucha, Christos Triantos

**Affiliations:** 1 Division of Gastroenterology, Department of Internal Medicine, University of Patras, 26504 Patras, Greece; iaggel@upatras.gr (I.A.); ploutarchosp96@gmail.com (P.P.); 2 Laboratory of Bioethics, School of Medicine, Democritus University of Thrace, 68100 Alexandroupolis, Greece; atsarouc@med.duth.gr

**Keywords:** irritable bowel syndrome (IBS), patient-centered care, ethical communication, therapeutic alliance, trust, shared decision-making, relational autonomy, self-management, nutritional management, dietary interventions, low-FODMAP diet

## Abstract

Irritable bowel syndrome (IBS) is a prevalent disorder of gut–brain interaction, characterized by a substantial symptom burden, impaired quality of life, and increased healthcare use. Despite advances in diagnostic criteria and treatment strategies, many patients feel dismissed, inadequately informed, or uncertain about the nature and meaning of their symptoms; these experiences may undermine trust and reduce engagement with healthcare professionals. The aim of this narrative review is to synthesize clinical and ethical considerations and propose a patient-centered ethical framework for IBS management, positioning communication as a core therapeutic intervention. We highlight how validation, clear and non-stigmatizing explanations, transparency about uncertainty, and recognition of patient values can strengthen the therapeutic alliance, support relational autonomy, and enable shared decision-making. These elements can promote supported self-management and improve adherence to individualized dietary, behavioral, and pharmacologic strategies. In response to the central role of nutrition in IBS care, we further integrate dietary management into the ethical framework, addressing dietary assessment, first-line dietary advice, soluble fiber, the structured low-FODMAP approach, and the risks of excessive or unsupported food restriction. We further discuss how the incorporation of patient-reported outcomes (PROs) can translate patient priorities into measurable outcomes, monitor clinically meaningful changes over time, and reduce discrepancies between clinical assessment and patients’ lived experiences. Finally, we underscore the impact of stigma and uncertainty and provide practical communication approaches to support a stronger therapeutic alliance in IBS care. The integration of ethical communication, PROs, and nutrition-sensitive self-management may improve patient experience, strengthen adherence, and support individualized therapeutic strategies in IBS care.

## 1. Introduction

Irritable bowel syndrome (IBS) is a prevalent, multifactorial disorder of the gastrointestinal tract, characterized by heterogeneous and fluctuating symptoms that may result in variable clinical presentations among patients [1,2]. Chronic or recurrent abdominal pain, bloating, altered bowel habits, and changes in stool consistency are among the most commonly reported symptoms in this population [1,2]. The global prevalence of IBS is estimated to be approximately 3–5% based on Rome IV criteria [3], whereas earlier studies using Rome III reported rates as high as 10% [3]. However, IBS prevalence can vary substantially across different geographic locations and is much higher among female individuals [4]. Recent mechanistic studies have shifted the focus from the assessment of gastrointestinal factors to broader biological and psychosocial interactions. During the past decade, functional gastrointestinal disorders including IBS have been redefined as disorders of gut–brain interactions (DGBIs), a group of conditions marked by chronic gastrointestinal symptoms that emerge from the interplay among visceral hypersensitivity, impaired gut motility, mucosal and immune dysfunction, dysbiosis of the gut microbiota, and dysregulated central nervous system (CNS) signaling [2,5].

The diagnosis of IBS is challenging due to variability in symptoms and their overlap with those of other gastrointestinal conditions [6,7]. IBS is classified into four different subtypes based on predominant symptoms: diarrhea-predominant IBS (IBS-D), constipation-predominant IBS (IBS-C), mixed-type IBS (IBS-M), and unclassified IBS (IBS-U) [1,8]. Symptoms are further shaped by psychosocial stressors and dietary exposure, both of which can increase clinical burden [9,10].

The combined effect of these biological, psychosocial, and developmental influences translates into a critical challenge for both patients and healthcare systems. IBS constitutes one of the most common reasons for referral to gastroenterology outpatient clinics and imposes a substantial burden on healthcare systems due to the frequent use of medical services and pharmacological interventions. The indirect economic consequences associated with this disorder are also of crucial importance, including absenteeism from work and reduced productivity [11]. Therefore, although IBS is often perceived as a benign condition, it imposes a substantial psychosocial and functional impact on patients, influencing daily routine, work productivity, social interactions, and psychological well-being, and overall significantly impairing health-related quality of life [12,13]. The complexity of IBS is further exacerbated by its uncertain etiology, relapsing–remitting symptoms, and the limited availability of validated disease-specific biomarkers, all of which complicate the interaction between patients and clinicians. These features make IBS a distinct ethical challenge in clinical practice. The absence of visible structural disease or definitive biomarker confirmation may increase diagnostic uncertainty, expose patients to symptom invalidation, and reinforce the perception that symptoms are not objectively verifiable. Together with social stigma and the intimate nature of bowel symptoms, these factors support the need for a dedicated patient-centered ethical framework [14,15].

These challenges highlight the imperative need to consider not only pathophysiological mechanisms but also the interpersonal and ethical dimensions of IBS care. Traditional clinical management was predominantly based on exclusion diagnostics and symptomatic treatment strategies, rather than mechanism-based interventions [16]. However, current evidence highlights the central role of non-pharmacological factors, such as quality of communication between clinicians and patients, decision-making autonomy, health literacy, self-management capacity, and the therapeutic alliance in determining clinical outcomes [16,17,18]. Importantly, empathy involves more than emotional awareness; it also includes taking time to think deliberately and reflectively, which often leads to a more accurate understanding than relying on quick impressions or stereotypes. This suggests that empathic skills are not fixed but can be learned and strengthened over time [19,20,21]. Lack of empathy during IBS care may lead to patient frustration, delay in diagnosis, minimization of symptoms and social stigmatization, and insufficient explanations of the condition [21]. These challenges are not only interpersonal, but primarily reflect deeper ethical concerns related to respect, transparency in communication, informed decision-making, and equitable access to care. Thus, an ethical framework is essential to guide more compassionate, effective, and patient-centered approaches to IBS management.

Nutrition represents a key link between pathophysiology, symptoms, and self-management in IBS. Many patients associate symptom fluctuations with meals and modify their dietary habits before or between clinical consultations. However, unsupported dietary experimentation may lead to inconsistent advice, unnecessary food avoidance, nutritional compromise, or food-related anxiety. Recent population-based data are consistent with this concern, showing that individuals with self-reported IBS were more likely than those without IBS to avoid gluten, lactose, white flour, and alcohol, with food avoidance largely driven by abdominal pain [22]. Moreover, diet-related worry and anxiety were significantly more prevalent among individuals with IBS, suggesting that dietary management is not only a nutritional issue but is also behavioral and psychological. Evidence-based nutritional management therefore requires more than a list of foods to avoid. It requires individualized dietary assessment, clear explanation of underlying mechanisms, realistic goals, and follow-up that helps patients identify tolerable patterns while preserving dietary diversity and quality of life [22,23,24,25]. In this context, dietary care extends beyond symptom control and becomes an ethical domain in which autonomy, beneficence, non-maleficence, and justice intersect.

The aim of this review is to synthesize clinical and ethical considerations to present a patient-centered ethical framework for IBS management, with a particular focus on how communication shapes trust, shared decision-making, supported self-management, and individualized care.

## 2. Autonomy as a Relational Capability

### 2.1. Relational Autonomy in IBS Care

Autonomy refers to the ability and right of individuals to make informed and voluntary choices, free from coercive influences, alongside the duty of others to respect those choices [26]. It constitutes a fundamental ethical principle in bioethics and healthcare. However, this classical view, which emphasizes independence and isolated decision-making, has proven insufficient in contemporary clinical practice. Relational autonomy is based on patient decision-making as a dynamic process shaped by interpersonal relationships, communication, and social context, rather than as an isolated individual choice [27]. Patient autonomy is closely associated with the quality of clinical communication, relational trust, and the recognition of patients’ lived experiences [28]. In parallel, the behavior of clinicians can strengthen or undermine autonomy by affecting self-perception, self-efficacy, and the individual’s ability to direct their own life. Within the context of IBS, autonomy becomes even more critical. Patients with IBS frequently report limited understanding of their diagnosis and difficulty interpreting their symptoms, especially when symptoms are attributed primarily to psychological distress. Recent evidence has highlighted that particularly among women with IBS, the disorder is often underdiagnosed or misdiagnosed, and patients commonly experience dismissal or minimization of their concerns, leading to feelings of invalidation, frustration, and psychological distress [9]. These experiences not only impair well-being but also weaken patients’ capacity to be confident and autonomous in the therapeutic process [9].

### 2.2. Continuity of Care and Therapeutic Relationships

In IBS, relational autonomy is closely connected to continuity of care. Because IBS symptoms are chronic, fluctuating, and often influenced by diet, stress, and daily routines, patients benefit from a stable therapeutic relationship in which concerns can be revisited over time [29]. Continuity allows clinicians to follow symptom patterns, evaluate treatment responses, adjust dietary or pharmacological strategies, and address emerging fears or misconceptions without restarting the explanatory process at every visit [29].

A sustained patient–clinician relationship also supports trust and shared decision-making. When patients feel known, believed, and consistently supported, they are more likely to disclose sensitive concerns, discuss treatment barriers, and participate actively in self-management [16,30,31]. Thus, continuity of care is not only a major feature of IBS management, but also an ethical condition that strengthens autonomy, reduces uncertainty, and helps transform IBS care into a collaborative long-term process [29].

Overall, relational autonomy can reshape IBS management by emphasizing how clinicians’ behavior, tone, explanations, empathy and consistency over time directly influence patients’ capacity to make informed decisions [32]. Supportive interaction strengthens autonomy by helping patients make sense of their symptoms and engage in management, whereas dismissive feelings may undermine self-efficacy, promote confusion, and weaken trust in healthcare professionals [32]. Thus, the application of relational autonomy in IBS care requires the development of a clinical model in which healthcare professionals actively listen, provide comprehensive and comprehensible information, discuss and validate the patients’ experiences, and involve them in decision-making [33]. In this way, autonomy becomes not merely an ethical concept but a dynamic capacity that is developed through therapeutic relationships.

In practical terms, relational autonomy can guide everyday IBS management by shifting decisions from simple patient choice to supported, contextualized decision-making [27]. For example, when discussing dietary therapy, clinicians should explore previous food restrictions, cultural eating habits, resources, fears, and daily routines before recommending a low-FODMAP diet or other dietary changes. Similarly, treatment selection should integrate symptom profile, patient priorities, previous experiences, concerns about medication, and willingness to consider behavioral or psychological strategies. Collaborative symptom monitoring, such as time-limited food and symptom diaries or PRO-based follow-up, can further support autonomy when used to identify patterns and guide shared decisions, rather than to place responsibility for symptom control entirely on the patient.

### 2.3. Ethical Limits of Autonomy in IBS Care

Autonomy in IBS care also has ethical limits. Respecting patient concerns does not mean that every request for further investigation should be followed when diagnostic criteria are fulfilled and alarm features are absent [29]. In patients who repeatedly request additional testing, clinicians should validate fear and uncertainty, explain why further investigations are not currently indicated, clarify which warning signs would justify reassessment, and offer structured follow-up [29]. This approach preserves trust and autonomy while avoiding unnecessary, low-value, or potentially harmful investigations.

## 3. Communication as an Ethical Intervention in IBS Management

### 3.1. Communication Deficits in IBS Care

IBS patients consistently report deficient communication, including limited explanation of their diagnosis, absence of empathy, limited opportunities to discuss their symptoms, and minimal involvement in decision-making [31]. The implications of this unsuccessful communication go beyond patient dissatisfaction, contributing to delays in diagnosis, use of unnecessary diagnostic procedures, poor treatment adherence, and diminished patients’ quality of life [31,34]. In contrast, effective patient–clinician communication is associated with better symptom control, lower healthcare use, and improved clinical outcomes, underscoring the importance of individualized communication strategies in IBS care [31].

The ethical significance of communication in IBS care has been reported in a focus group study exploring patient preferences for healthcare delivery [35]. Masclee et al. found that patients consistently emphasized the need for clear and empathetic communication, to feel that healthcare staff have taken them seriously, and to understand that IBS is a real condition even without a definitive cure [35]. The same study showed that patients prefer multidisciplinary care, including gastroenterologists, dietitians, nurses, and psychologists ideally organized in centers of expertise, with a step-by-step, individualized treatment plan [35]. It also emphasizes the need for reliable, accessible information and stronger links between clinical care and ongoing scientific research, which together could improve coping, quality of life, and reduce unnecessary healthcare use [35].

**Figure 1:**
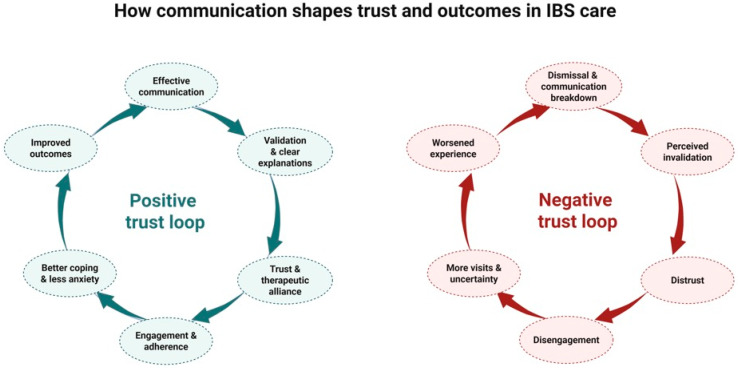
Trust loops in irritable bowel syndrome (IBS) care. Effective communication can initiate a positive feedback loop leading to validation, trust, engagement, and improved outcomes, whereas dismissive communication may trigger a negative loop characterized by invalidation, distrust, disengagement, and worsening patient experience. Created in BioRender. Aggeletopoulou, I. (2026) https://app.biorender.com/illustrations/696f7e75a3c6c8eff6265788?slideId=93d8f75b-2e8b-442a-a367-cc2dd66500d7 (accessed on 12 June 2026). Abbreviations: IBS, irritable bowel syndrome.

Effective IBS management is based on a strong patient–provider relationship characterized by trust, respect, and shared understanding [29]. The British Society of Gastroenterology guidelines emphasize that such relationships improve symptoms and quality of life while reducing healthcare utilization and supporting treatment adherence [29]. During the consultation process, the establishment of early engagement is of crucial importance and should involve the development of a collaborative dynamic in which clinicians and patients work together for a common goal. Simple non-verbal behaviors, including sustained eye contact, open posture, and body language, play a substantial role in developing honest communication [36]. The impact of communication on trust and clinical outcomes in IBS can be depicted as self-reinforcing feedback loops (Figure 1).

### 3.2. Building Therapeutic Alliance: Empathy and Validation

Empathy is central to effective communication in IBS care, as it allows clinicians to understand the patient’s experience while at the same time maintaining professional objectivity [21]. Unlike sympathy, empathy involves the recognition of patients’ distress without becoming emotionally involved, thus helping to build trust between patients and providers [37]. Recent evidence that explored patients’ and their relatives’ experiences of empathic and compassionate healthcare encounters highlighted communication behaviors, clinician attitudes, and small gestures as key determinants of patient experience and outcomes [38].

Validation is closely linked to empathy and is particularly critical in IBS, where patients frequently feel dismissed [24,31]. Recognizing that symptoms are real and meaningful helps patients feel understood and encourages open communication and shared decision-making. Together, empathy and validation create a therapeutic environment in which patients can engage in therapeutic decision-making.

### 3.3. Explaining IBS to Patients as a Disorder of Gut–Brain Interaction

Advances in IBS research have redefined the condition as a DGBI rather than a functional disorder, offering clinicians a clearer explanatory model [2]. The British Society of Gastroenterology guidelines recommend that clinicians explain this concept using simple, non-technical language, emphasizing the continuous communication between the gut and brain and how factors such as diet, stress, and emotional responses can disrupt this signaling and produce symptoms [29]. This approach allows the maintenance of scientific accuracy while at the same time improving patient understanding.

The first step in effective patient education about IBS is to assess patients’ existing beliefs and understanding of their condition [31]. The identification of prior knowledge allows clinicians to address misconceptions, determine the patient’s level of understanding, and adapt explanations accordingly, while also maintaining respect for the patient’s opinions and perspective [31]. This approach is particularly important for the correction of common misunderstandings, such as fears of disease progression, misattribution to food allergies, or the belief that symptoms are all in the mind. When clinicians respond to patient misconceptions, they should combine accurate information with the validation of patient concerns. Instead of dismissing patients’ fears, clinicians can build reassurance and trust by acknowledging their worries and clearly explaining how IBS differs from inflammatory or malignant conditions [31]. Emphasizing that IBS involves functional and sensory alterations, despite the absence of structural abnormalities, helps patients to validate their symptoms while directly addressing common fears about disease progression.

### 3.4. Discussing Psychological Factors with Sensitivity

Ιn IBS, the discussion of psychological factors requires particular sensitivity, as patients may perceive reports to stress as implying that their symptoms are not real [21]. An effective strategy for the clinician is to first acknowledge that chronic gastrointestinal symptoms can themselves promote stress and emotional burden, thereby validating the patient’s physical experience [31]. Healthcare professionals can then introduce the gut–brain axis as a bidirectional biological and molecular pathway through which emotional states, stress reactivity, visceral sensitivity, and intestinal function interact [39]. Within this explanation, psychological support, cognitive behavioral therapy (CBT), and mindfulness-based strategies can be presented as tools that target gut–brain regulation, reduce symptom-related fear and hypervigilance, and improve coping [25,34]. Similarly, physical activity should be communicated as a supportive and individualized self-management strategy rather than as a simple lifestyle prescription [40,41]. Clinicians may assess activity levels and encourage gradual increases in movement when appropriate, while acknowledging that fatigue, pain, symptom variability, and patient preferences may limit what is feasible [40,41]. Framing these interventions as part of integrated IBS care, rather than as evidence that symptoms are only psychological, may reduce stigma and increase patient acceptance.

### 3.5. Structural and Cultural Barriers to Effective Clinician–Patient Communication

The limited duration of clinical encounters constitutes a significant barrier to comprehensive IBS education, as a substantial number of clinic visits is dedicated to administrative tasks rather than direct interaction with the patient. Momenipour et al. reported that direct face-to-face interaction accounts for only about 40–50% of a typical clinic visit, restricting the available time for meaningful conversation [42]. Further study indicates that patients with severe IBS often experience healthcare encounters as dismissive, which can lead to confusion, self-doubt, and a need to justify their symptoms [43]. In contrast, feeling listened to, believed, and taken seriously is a key part of supportive, patient-centered care [43].

To address this challenge, clinicians should prioritize key concerns during initial visits and build understanding over the course of follow-up visits [31]. Beyond in-person discussions, providing patients with written resources, trusted online material, or structured educational tools can further enhance patient comprehension while reducing the need for face-to-face time [31]. A further approach to improve time efficiency is the use of group-based education sessions, in which clinicians present core IBS information to several patients at once, allowing individual consultations to focus more specifically on individualized management and specific concerns of each patient [44]. Additionally, short follow-up telephone calls or secure digital messaging can help address questions that emerge after the visit, thereby maintaining continuity of care and enhancing the overall effectiveness of in-person appointments.

### 3.6. Ethical Implications of Communication in IBS Management

Research evidence demonstrates that the way clinicians present information can substantially influence patients’ understanding and acceptance of their diagnosis. To communicate effectively, clinicians can use a few evidence-informed phrases and adapt them to their own style and to what each patient needs. Data from telehealth further demonstrates that communication challenges are shaped not only by individual clinician behavior, but also by contextual factors, pointing out the need for structured, patient-centered communication practices [45]. Notably, recent evidence also showed that these communication challenges are not exclusively the result of individual clinician behavior but are strongly shaped by the culture of the clinical unit itself [46]. An observational study analyzing real-life hospital encounters found that nearly 10% of variation in physicians’ communication framing was explained by unit-level patterns, whereas individual differences accounted for less than 1% [46]. Gastroenterology units, where the majority of patients with IBS are managed, demonstrated a distinct communication profile characterized by a greater use of inviting and convincing expressions and a tendency to emphasize what the clinician considered important, rather than expressions that support reassurance or shared understanding [46]. Such specialty-specific patterns can make IBS patients feel dismissed or pushed, especially those who already live with uncertainty and stigma [46].

**Figure 2:**
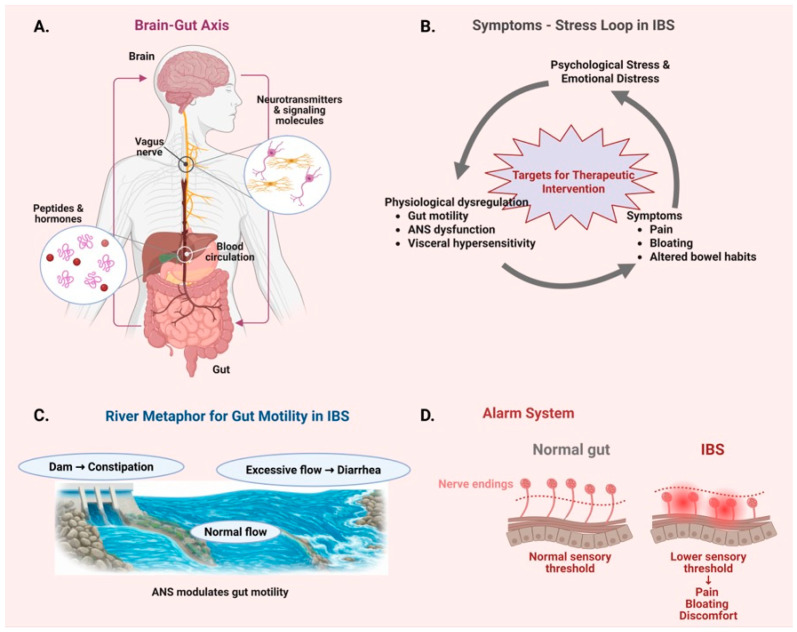
Patient-friendly conceptual framework for understanding irritable bowel syndrome (IBS). (**A**). Schematic overview of bidirectional communication between the central nervous system and the gastrointestinal tract through neural, hormonal, and circulatory pathways. (**B**). Representation of the vicious cycle between psychological stress, physiological dysregulation, and gastrointestinal symptoms. (**C**). Metaphorical illustration of intestinal transit as a flowing river, where altered regulation leads to reduced or accelerated movement. (**D**). Comparison of normal visceral sensory signaling with heightened sensitivity in IBS, illustrating lower activation thresholds associated with pain and discomfort. Created in BioRender. Aggeletopoulou, I. (2026) https://app.biorender.com/illustrations/696f7e75a3c6c8eff6265788?slideId=83afea59-e991-4c4b-8c29-2ce8a5aa1b80 (accessed on 12 June 2026). Abbreviations: IBS, irritable bowel syndrome; ANS, autonomic nervous system.

In this context, structured communication approaches have become especially important. When clinicians introduce the diagnosis, they may emphasize that IBS is a recognized medical condition caused by changes in gut function and gut–brain signaling, and that although symptoms may be challenging, the disorder does not lead to intestinal injury or serious disease [29]. In order to explain gut–brain interaction, it can be helpful to describe it as a bidirectional communication network that becomes overresponsive in IBS, producing real physical sensations rather than symptoms that are ‘psychological’ in origin [47]. When addressing psychological influences, clinicians can validate the patient’s distress and clarify how stress may trigger or exacerbate symptoms through neural pathways [48]. It is also of crucial importance to emphasize that this cycle can be interrupted through symptom-directed treatments and interventions that aim to reduce stress response [48]. Visual explanatory models can support this process by translating complex gut–brain interactions into accessible frameworks (Figure 2).

These communication challenges carry significant ethical weight, as they influence how patients interpret their condition, understand their therapeutic options, and ultimately develop trust in the healthcare system [49]. Evidence from studies on clinician–patient communication in functional gastrointestinal disorders showed that inadequate communication contributes to internalized stigma, reduced adherence to therapy, heightened anxiety, and overall dissatisfaction with care [15,50]. Poor communication may leave patients feeling disbelieved, uncertain about treatment goals, or abandoned, while also reinforcing misconceptions, such as the belief that IBS is “purely psychological” [15,50].

Population-based evidence further demonstrates the impact of these communication failures. Olafsdottir et al. reported that only half of the patients who asked for medical consultation for IBS symptoms received a diagnosis, just 12.8% were satisfied with their treatment, and 43% reported that IBS significantly impaired their daily activities [51]. Moreover, physicians rarely applied standardized diagnostic criteria, with only 22.5% using the Rome III IBS diagnostic criteria, contributing thus to inconsistent explanations and uncertainty during the clinical course [51]. These findings highlight how inadequate communication and diagnostic ambiguity undermine respect and transparency in IBS care.

Effective communication is a key part of IBS management. Clear and structured explanations of the diagnosis and the gut–brain axis, normalization of symptom variability, transparent discussion of realistic expectations, and the use of open-ended questions help patients express concerns and develop a more coherent understanding of their condition. Furthermore, the validation of the patient’s experience and the incorporation of shared decision-making into clinical encounters is very important. Research on communication in IBS demonstrates that these practices strengthen trust, reduce uncertainty, and promote patient engagement, while patient preference studies highlight that individuals with IBS value clinicians who communicate respectfully and acknowledge the multifactorial nature of the disorder. Importantly, the quality of the patient–clinician relationship itself has been shown to influence clinical outcomes in IBS, underscoring communication as an integral component of treatment rather than merely supportive care. Framing IBS within a gut–brain interaction model further provides a rational explanatory approach that combines physiological and psychological processes without undermining the reality of symptoms. Communication should also address misinformation in a non-confrontational way. Clinicians should explore what patients have read or believe, validate the concern, and provide clear evidence-based explanations to reduce confusion, unnecessary restrictions, and repeated requests for low-value investigations [41,52]. Ultimately, communication is not a peripheral element of care; it is an ethical practice that can profoundly shape how patients experience their symptoms and engage in self-management. This ethical approach is especially relevant to dietary communication, where unclear or overly restrictive advice may reinforce food-related fear, unnecessary avoidance, and the perception that patients are solely responsible for controlling their symptoms.

## 4. Patient-Reported Outcomes in IBS

### 4.1. Scope and Practical Challenges

Patient-reported outcomes (PROs) are primarily assessed through standardized questionnaires designed to evaluate multiple dimensions of the patient experience, including physical symptoms, psychological factors, and social functioning [53,54]. In IBS, PROs offer valuable insights into disease burden from the patient’s perspective and have the potential to strengthen clinician–patient communication and support shared decision-making [55]. Systematic PRO collection has been shown to improve patient satisfaction and promote the patient–provider relationship [55]. Evidence suggests that IBS PRO scores reflect not only gastrointestinal symptom burden but also broader aspects of health and well-being [56]. Psychological factors such as somatization, pain catastrophizing, and anxiety sensitivity can strongly influence how patients experience and report their symptoms, helping explain a meaningful part of the variation seen in commonly used PRO measures [56]. As a result, global and composite PROs may overrepresent pain-related distress while underestimating other major IBS features, such as bowel habit alterations [56].

Beyond their measurement function, PROs also have ethical significance in IBS care. By formally incorporating the patient’s voice into clinical assessment, PROs can make symptoms, treatment burden, quality-of-life impairment, and psychosocial concerns more profound during decision-making [53,54,55]. This is particularly important in IBS, where the absence of definitive biomarkers may lead to the underestimation of patient suffering [14,15]. When used appropriately, PROs can support relational autonomy by helping patients express priorities, monitor changes over time, and participate more actively in shared decisions [27,55]. They may also reduce paternalistic approaches by shifting clinical evaluation from clinician-centered interpretation alone toward a more balanced understanding of the patient’s lived experience. In this way, PROs can contribute to more equitable care, especially for patients whose symptoms are minimized, poorly explained, or not fully captured during brief consultations.

However, there are several barriers that limit their routine integration into clinical practice [57]. The administration and interpretation of PRO instruments may be considered time-consuming and demanding, particularly in clinical settings where data must be incorporated into electronic health records [58]. In addition, many clinicians lack formal training in biopsychosocial assessment frameworks, making it challenging to interpret PRO scores and translate them into effective management strategies [58]. The availability of numerous PRO instruments, combined with the absence of uniform measurement standards, further complicates their clinical use [58]. Moreover, evidence from clinical trials indicates that the collection of heterogeneous IBS symptom domains into a single severity score can restrict differential treatment effects across various symptom domains, thereby limiting the interpretability and sensitivity of PRO-based endpoints [59]. As a result, symptom assessment in everyday practice often relies on informal evaluation rather than structured PRO measures.

### 4.2. Specific Patient-Reported Outcome Instruments for IBS Patients

A wide range of IBS-specific PRO instruments have been developed to assess symptom severity and health-related quality of life. These PROs can vary in structure, length, and degree of validation. Commonly used symptom-focused tools include global measures such as the Adequate Relief measure [60], as well as multidimensional instruments like the IBS Severity Scoring System [61]. In parallel, several IBS-specific health-related quality-of-life questionnaires have been designed to describe the impact of the disorder on daily functioning and well-being [62,63,64,65]. Comparative assessment of these instruments suggests that brief global measures are suitable for assessing overall symptoms, whereas more detailed scales provide greater insight into specific symptom domains [66]. Among the quality-of-life tools, the IBS Quality of Life questionnaire remains the most extensively validated for detecting major clinical changes 65, 66]. Despite their methodological strengths, many IBS-specific PRO instruments are lengthy and difficult to implement in daily routine and are therefore considered impractical for everyday clinical practice. To provide a practical overview of available IBS-specific PRO instruments, Table 1 summarizes their main domains, clinical strengths, and limitations in routine care.

These limitations resulted in the development of the Patient-Reported Outcomes Measurement Information System (PROMIS) [67]. The National Institutes of Health PROMIS was designed to provide a standardized, flexible framework for assessing patient-reported health outcomes across various conditions. By using modern psychometric tests and digital administration, PROMIS enables efficient and meaningful PRO assessment while emphasizing the critical role of the patient’s perspective in evaluating health and treatment [67]. In the context of gastrointestinal disorders, PROMIS was expanded to include gastrointestinal symptom-focused items [68]. These gastrointestinal PROMIS scales were developed to assess key digestive symptoms, such as abdominal pain, bloating, bowel habits, and nausea, by using a symptom-based rather than disease-specific approach, making it applicable to various gastrointestinal conditions, including IBS [68]. The scales enable standardized symptom quantification, comparison with population data, and longitudinal monitoring, thereby supporting patient engagement, treatment evaluation, and improved communication between patients and clinicians.

Among the domains captured by patient-reported outcomes, diet-related symptoms and food-related concerns are particularly important in IBS care. Many patients monitor their symptoms in relation to meals and frequently modify their diet before receiving structured clinical guidance. Therefore, nutritional management represents a key area where patient-reported experience can directly inform shared decision-making and ethically supported self-management.

## 5. Nutritional Management in IBS: Dietary Modification Within a Patient-Centered Therapeutic Framework

### 5.1. Diet as a Therapeutic and Ethical Domain in IBS Care

Dietary management constitutes a particularly practical and personally meaningful aspect of IBS care, given that food-related decisions occur daily, are shaped by family and cultural contexts, and are often interpreted by patients as reflecting either control over, or loss of control in relation to, their symptoms [69]. For many patients, meals are associated with abdominal pain, bloating, diarrhea, constipation, or fear of symptom exacerbation in public settings. These experiences may encourage self-directed elimination diets, repeated food avoidance, or reliance on unverified information from social media and commercial sources. Importantly, symptom-driven restriction may become self-perpetuating, as fear of postprandial gastrointestinal symptoms can reinforce avoidance, reduce dietary variety, and contribute to nutritional and psychosocial burden [70].

The psychological dimension of eating behavior is particularly important in IBS [71]. In IBS, food-related symptoms may lead patients to skip meals, avoid specific foods, or adopt restrictive eating patterns in an attempt to prevent bloating, abdominal pain, or diarrhea [72]. Although dietary modification can be therapeutically useful, unstructured restriction may increase fear of eating, reduce social participation, and make patients feel personally responsible for symptom control [73]. Clinicians should therefore explore not only which foods patients avoid, but also why they avoid them, how dietary restriction affects daily life, and whether food-related fears are becoming disproportionate or harmful [72].

A patient-centered ethical framework should therefore move beyond the idea that dietary management is merely a matter of willpower or compliance and instead recognize the lived challenges patients face when food becomes linked to symptoms, uncertainty, and fear. Clinicians should therefore use diet as a shared therapeutic domain that requires validation of the patient’s experience, assessment of prior dietary attempts, identification of values and practical constraints, and protection from harm [74]. 

A nutrition-sensitive consultation should begin with a structured but non-judgmental dietary history. Clinicians can explore meal timing, portion size, hydration, caffeine and alcohol intake, fat intake, fiber sources, use of sugar alcohols, lactose- or fructose-containing foods, ultra-processed foods, previous exclusion diets, weight change, nutritional adequacy, and the extent to which symptoms affect social eating [75]. This assessment helps distinguish actual dietary contributors to reproducible symptoms from broad and unnecessary restrictions. It also provides an opportunity to correct misconceptions, such as the belief that all food-related symptoms represent allergy or permanent intolerance, while still acknowledging that dietary triggers can generate real physical symptoms through osmotic effects, fermentation, gas production, changes in motility, and visceral hypersensitivity [76].

### 5.2. Evidence-Based Dietary Interventions and Personalization

General dietary and lifestyle advice usually represents the first nutritional step. This may include regular meals, avoidance of very large meals, adequate hydration, moderation of caffeine, alcohol, and high-fat meals, and attention to foods that reproducibly worsen symptoms. Soluble fiber is supported for the management of global IBS symptoms and is particularly relevant when constipation is prominent, whereas excessive or abrupt intake of poorly tolerated fiber may worsen bloating and discomfort [77]. For this reason, psyllium or other soluble fiber should be introduced gradually and titrated according to symptoms, tolerance, and stool pattern [23,25,29]. This practical approach supports autonomy by giving patients a safe, understandable, and reversible strategy rather than introducing multiple restrictions that make symptom interpretation difficult. For patients with persistent symptoms despite first-line advice, the low-FODMAP diet is currently considered the most evidence-based dietary intervention for IBS [78]. Its therapeutic rationale is based on reducing fermentable short-chain carbohydrates that can increase luminal water content and gas production, thereby contributing to distension and symptom generation in susceptible patients. Importantly, the low-FODMAP diet should be framed as a structured, time-limited therapeutic trial rather than a permanent restrictive eating pattern. The recommended process includes three phases: short-term restriction, usually for no more than 4 to 6 weeks; systematic reintroduction of FODMAP groups to identify tolerance thresholds; and long-term personalization aimed at the least restrictive diet compatible with symptom control [23,79]. Patients who do not experience meaningful benefit during the restriction phase should be supported to discontinue the intervention and consider alternative therapeutic options rather than continuing unnecessary dietary limitations.

Personalization is ethically important because the ultimate goal of dietary therapy is not maximal restriction but sustainable symptom control, nutritional adequacy, and preservation of everyday life. Long-term data suggest that after structured restriction, reintroduction, and personalization, a substantial proportion of patients report adequate symptom relief while some microbiota-related effects of short-term restriction may be mitigated [80]. Conversely, prolonged restriction may reduce dietary variety and diet quality, may affect the intake of fiber and micronutrients, and may influence the gut microbiota [81]. Therefore, patients should be informed from the beginning that reintroduction and personalization are essential parts of dietary treatment, not optional steps to be considered only once symptoms improve [24,79].

Other dietary approaches, including gluten-free or lactose-restricted diets, should be discussed carefully and individualized according to clinical context. Although some patients report benefit from gluten avoidance, randomized trials have yielded mixed results, and improvement may reflect a reduced intake of wheat fructans rather than gluten itself [23,82]. Similarly, lactose restriction is most appropriate when symptoms are reproducibly related to lactose-containing foods or when lactose malabsorption is clinically suspected [23]. This distinction is ethically important because labeling foods as harmful without clear evidence may increase fear, social burden, and unnecessary long-term restrictions.

### 5.3. Safety, Equity, and Shared Decision-Making in Dietary Care

Dietary interventions in IBS can also produce harm if implemented without support. Restrictive diets may be inappropriate for patients with unexplained weight loss, low body mass index, malnutrition, eating disorders, severe food-related anxiety, inability to understand or apply the diet safely, or food insecurity [72]. These considerations should not be interpreted as reasons to withhold dietary therapy from patients who may benefit. Instead, they underscore the importance of adapting dietary interventions to the patient’s clinical, nutritional, and psychological context, with dietitian support and close follow-up when needed [24,79]. From an ethical perspective, recommending a complex diet without assessing literacy, resources, culture, cooking facilities, work schedule, family responsibilities, and access to suitable foods may unintentionally shift the burden of care onto the patient and widen inequalities. The economic and organizational burden of dietary care also has ethical relevance. Specialized diets often require more time, money, and access to experienced dietitians, resources not equally available to all patients [83]. Ethical nutritional care should therefore adapt recommendations to the patient’s circumstances and avoid assuming that complex dietary interventions are universally feasible [83].

**Figure 3:**
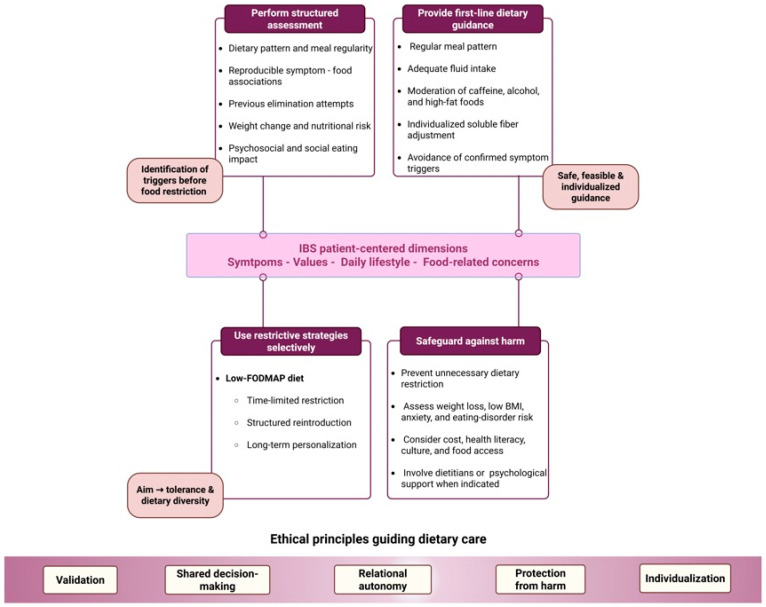
Nutrition-sensitive, patient-centered dietary management in irritable bowel syndrome. Figure 3 summarizes a structured approach to dietary care in IBS, including nutritional assessment, first-line dietary guidance, selective use of restrictive strategies, and measures to prevent nutritional, psychological, and social harm. Patient symptoms, values, lifestyle, and food-related concerns remain central throughout the process. Created in BioRender. Aggeletopoulou, I. (2026) https://app.biorender.com/illustrations/696f7e75a3c6c8eff6265788?slideId=2abe8ce2-7956-41fb-aa69-da2341cd15a1 (accessed on 12 June 2026). Abbreviations: BMI, body mass index; IBS, irritable bowel syndrome.

Shared decision-making can transform dietary therapy from an obligation into supported self-management. Clinicians should explain the expected benefits, uncertainties, time frame, and stopping rules of each dietary option. They should ask what level of change the patient considers feasible and agree on simple monitoring targets [84]. Food and symptom diaries may be useful when they are time-limited and focused on learning, but they should not become a source of guilt, hypervigilance, or excessive symptom checking [85]. PROs can complement dietary follow-up by capturing abdominal pain, bloating, stool pattern, quality of life, food-related anxiety, and treatment burden [86]. Thus, dietary modification becomes integrated into the wider ethical framework of IBS care by respecting patient values, supporting autonomy through understandable choices, protecting against nutritional and psychological harm, and maintaining the therapeutic alliance. Figure 3 summarizes the proposed nutrition-sensitive, patient-centered approach to dietary management in IBS.

## 6. Self-Management in IBS: Autonomy, Responsibility, and Ethical Limits

Given the limitations of pharmacological treatments and standardized outcome measures, IBS care increasingly relies on patients’ active participation in daily symptom management, including dietary modification, symptom monitoring, behavioral strategies, and treatment adherence. In this context, self-management emerges as a cornerstone of IBS management, not only as a behavioral strategy, but as an ethically grounded practice closely linked to the principles of autonomy, justice, and beneficence [87]. The nutrition-sensitive approach described above illustrates why self-management must be supported rather than delegated to patients as individual responsibility alone. Evidence from structured self-management interventions in IBS suggests sustained use; approximately 90% of participants continued to use multiple self-management strategies one year after program completion, with adherence rates above 79% in every subtheme [88]. Notably, the benefits were not related to any single technique, but rather to the flexibility of the intervention and the patients’ ability to select strategies aligned with their individual needs [88]. Taken together, these findings support self-management as an ethically grounded approach that promotes autonomy and responsibility while recognizing that not all patients are able to engage to the same extent.

The ethical importance of self-management in IBS can also be understood through established behavioral concepts. Self-efficacy theory emphasizes that patients are more likely to initiate and maintain health-related behaviors when they feel capable of performing them [89], while self-care and chronic disease self-management models highlight the importance of problem-solving, action planning, communication with healthcare professionals, and confidence-building [90,91]. In IBS, these concepts support the view that self-management should not be interpreted as a transfer of responsibility to the patient, but as a supported process shaped by information, skills, therapeutic trust, and access to care [89,90,91]. Evidence from the IBD literature, where self-care, self-efficacy, and patient engagement have been extensively studied, provides useful theoretical support for understanding how long-term gastrointestinal care can combine patient empowerment with structured professional guidance [92].

### Effects of Digital Health Interventions on IBS Patient Outcomes

Digital health interventions (DHIs) comprise a broad range of technologies, including mobile applications, web-based platforms, telemedicine tools, and wearable systems, designed to support symptom monitoring, education, and self-management in individuals with chronic conditions [93]. In IBS, the interest in DHIs has expanded, with studies increasingly focusing on clinical benefit and patient-centered outcomes rather than feasibility alone. As DHIs become more common in IBS care, it is of crucial importance to evaluate their impact on patient-centered outcomes in order to validate whether they genuinely support empowerment and self-management. Overall, DHIs are associated with improvements in IBS symptom severity and health-related quality of life, findings consistent with evidence from DHIs applied in other chronic gastrointestinal and pain-related conditions [94,95]. Benefits are more consistently observed for symptom severity and quality-of-life outcomes, whereas effects on psychological distress are smaller and more heterogeneous across studies, and remain insufficiently characterized in IBS-specific digital interventions [96,97,98,99]. In pooled analyses of eHealth interventions across chronic gastrointestinal illness, small but statistically significant improvements have been reported for both quality of life (d = 0.25, *p* = 0.008) and psychological distress (d = 0.24, *p* = 0.017), suggesting a modest signal that may not translate uniformly across IBS-focused trials [100]. This variability suggests that not all therapeutic targets are equally responsive to digital delivery.

Current guidelines recommend brain–gut behavioral therapies (BGBTs) as effective adjunctive treatments for IBS [101]. However, access is often limited due to a lack of trained gastrointestinal psychologists, time constraints, and high cost. These barriers have accelerated the development of digital therapeutics aiming to deliver evidence-based approaches, such as CBT and gut-directed hypnotherapy [101]. DHIs that combine brain–gut behavioral components (e.g., CBT, gut-directed hypnotherapy, mindfulness-based approaches) with symptom or health monitoring have been more consistently associated with favorable outcomes in IBS, including improvement in symptom severity and quality of life [87,101]. In contrast, evidence supporting the effectiveness of diet-based DHIs is less consistent, despite the central role of dietary management in IBS care [95]. The impact of diet-focused DHIs also remains insufficiently characterized, particularly with respect to psychological outcomes, including food-related fear, gastrointestinal-specific anxiety, and pain catastrophizing [95].

Although IBS management is commonly framed as requiring personalized care, current evidence does not demonstrate a robust association between higher levels of DHI personalization and improved effectiveness outcomes [95]. More customizable or adaptive DHIs have not consistently outperformed simpler, less personalized interventions in terms of symptom severity or quality of life [95]. These findings suggest that personalization itself may be insufficient to provide clinical benefits. Effectiveness appears to be more influenced by the therapeutic focus of the intervention than the degree of personalization. DHIs focused on brain–gut behavioral skills often demonstrate favorable outcomes across personalization levels, suggesting that intervention content and underlying mechanisms may outweigh customization in determining clinical benefit [95].

D’Silva et al., in their meta-analysis, did not demonstrate a significant difference in effectiveness outcomes between self-directed DHIs and hybrid interventions incorporating patient–provider communication [95]. These findings contrast with the broader literature highlighting the value of patient–provider interaction in chronic disease management, suggesting that the benefits of such communication may not be fully represented within current DHI designs or outcome measures [15].

Feasibility outcomes indicate that while DHIs are generally considered usable and acceptable, sustained engagement remains inconsistent [95]. This challenge between initial acceptability and long-term use represents a critical limitation for digital self-management strategies. More structured, behavior-based interventions, especially non-personalized brain–gut behavioral therapies, often demonstrate higher loss to follow-up despite favorable clinical outcomes. This may underscore a potential gap between short-term improvement and long-term adherence [95]. These findings highlight that clinical effectiveness does not necessarily translate into sustainable patient engagement. Taken together, evidence from early telemedicine and mobile health interventions in IBS suggest improvements in symptom severity, quality of life, and patient satisfaction, while at the same time revealing substantial variability in engagement and adherence. This pattern indicates that self-management should be viewed as a process that requires support and should be adapted to the patient’s context, rather than assumed to be feasible for all patients.

Recent mechanistic evidence further complicates the ethical framing of self-management in IBS by demonstrating that behavioral interventions may induce symptom-specific biological effects rather than uniform physiological improvement [102]. Multi-omics data from a nurse-led, person-centered self-management intervention in young adults with IBS indicate that pain improvement aligns with the attenuation of core gut microbial metabolic pathways, whereas anxiety-related outcomes align with changes in host transcriptomic pathways related to cellular energy metabolism [102]. In contrast, immune–inflammatory modules show limited regulation within the same intervention period [102]. These findings suggest that responsiveness to self-management is restricted by the underlying pathophysiology and varies across symptom domains. From an ethical perspective, these data suggest that patient engagement in self-management does not necessarily lead to uniform clinical improvement. Overall, the biological differences observed imply that patients may not achieve the same outcomes despite comparable effort. This supports the view that self-management should be supported, rather than treated as individual responsibility alone.

Engagement with DHIs appears to be modulated by multiple interacting factors beyond design of intervention, such as psychological burden, digital health literacy, motivation, cost, and alignment with patients’ daily routines [103,104]. Such factors may affect individuals with higher symptom burden to a greater extent, raising ethical considerations regarding equity and access. As most evidence derives from controlled research environments, the generalizability of feasibility findings to real-world IBS care remains limited.

Interpretation of these associations is further complicated by the multifunctional nature of many DHIs, which often integrate multiple therapeutic targets within a single intervention. As a result, evaluations that attempt to isolate single components may overlook synergistic effects. This highlights the need for more integrated assessment approaches to better understand how specific design features contribute to effectiveness.

At the same time, digital self-management tools introduce important ethical challenges. Concerns related to data privacy, unequal access to technology, and variations in digital literacy may exacerbate existing health disparities if not adequately addressed [105,106,107]. In this context, the ethical promise of self-management cannot be taken for granted, because some patients face barriers such as limited access, low digital skills, or high symptom burden.

Health literacy emerges as a critical determinant of whether self-management strategies translate into meaningful clinical benefit [108]. Low health literacy has been consistently associated with impaired decision-making, reduced treatment adherence, lower self-management capacity, and poorer health outcomes [108]. These findings emphasize that empowerment is not guaranteed by providing information or tools, but depends on supported engagement, understanding, confidence, and feasibility. In hospitalized patients, inadequate health literacy has been linked to a threefold increase in emergency department revisits within 90 days of discharge compared with adequate health literacy, even after adjustment for education level, highlighting that access to information does not necessarily translate into effective self-management [109]. In the context of IBS, where symptom interpretation and sustained behavioral engagement are central to care, limited health literacy may reduce the effectiveness of digital interventions and widen existing inequalities in outcomes.

Recent assessments of artificial intelligence-based patient education tools further underscore these ethical concerns. In a clinician-rated comparison of two widely used large language models for IBS education, 94.9% of Gemini-1 responses and 89.7% of ChatGPT-4 responses were comprehensive, with no statistically significant difference between models (*p* = 0.67) [110]. However, 7.7% of all responses were classified as “mixed”, containing vague, outdated, or partially incorrect information, most frequently in domains central to self-management such as general disease understanding and lifestyle factors [110]. Despite adequate accuracy, both models generated college-level text, with low Flesch Reading Ease scores (Gemini-1: 35.8 ± 3.3; ChatGPT-4: 32.3 ± 5.6), indicating poor suitability for patient education [110]. In terms of emotional engagement, ChatGPT-4 responses were rated as moderately empathetic in 100% of evaluations, whereas 66.7% of Gemini-1 responses were rated as only minimally empathetic [110]. These findings suggest that while AI-based educational tools can provide extensive information, they may disproportionately benefit patients with higher health and digital literacy, potentially widening existing informational gaps. The ethical value of digital self-management therefore depends not only on providing information, but on ensuring that it is accessible, understandable, and truly supportive of patient autonomy.

Accordingly, clinicians have an ethical duty to consider patients’ health and digital literacy, adapt information to the individual, check understanding, and direct patients to reliable and accessible educational resources. Self-management strategies that overlook differences in literacy, access, and cognitive burden risk shifting responsibility onto patients without providing the support they need to succeed.

## 7. Conclusions

**Figure 4:**
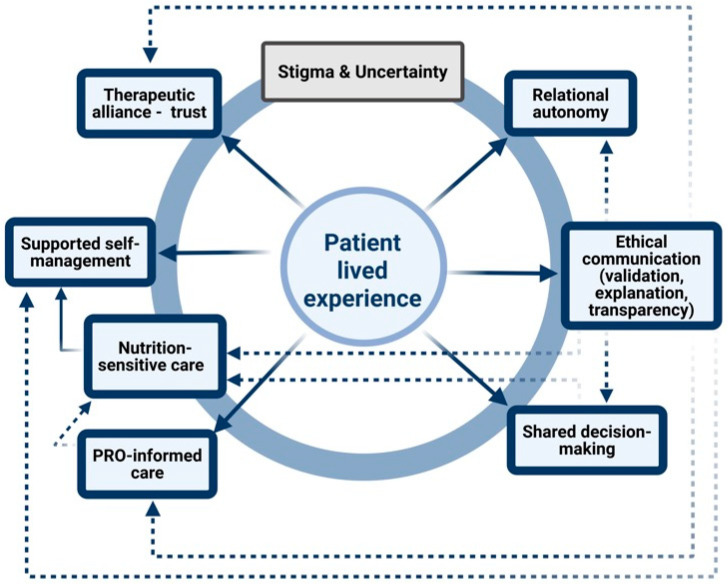
Patient-centered ethical framework for irritable bowel syndrome (IBS) care. The patient’s lived experience is placed at the center of care. Solid arrows indicate the core care domains, while dashed arrows show how ethical communication supports their integration. Stigma, uncertainty, and nutrition-sensitive self-management are incorporated as key factors shaping trust, autonomy, and shared decision-making. Created in BioRender. Aggeletopoulou, I. (2026) https://app.biorender.com/illustrations/696f7e75a3c6c8eff6265788?slideId=1987253c-32ec-4c13-8391-c5581bc72978 (accessed on 12 June 2026). Abbreviations: PROs, patient-reported outcomes.

IBS is a condition in which clinical outcomes are shaped not only by medical and pharmacological interventions, but also by the ethical quality of care, including trust, empathy, respectful communication, and nutrition-sensitive support. Since long-term management depends on patient engagement and shared decision-making, compassionate and accessible support becomes a key determinant of clinical outcomes. The absence of definitive biomarkers, the relapsing/remitting symptomatology, and the persistent stigma place patients at a high risk of diagnostic uncertainty. A patient-centered ethical framework for contemporary IBS management should therefore translate relational autonomy into routine clinical practice, including listening and validation, clear explanations framed within the gut–brain interaction model, individualized dietary counseling, and shared decision-making that explicitly integrates patients’ priorities and lived experience. Figure 4 summarizes the proposed framework.

Within this framework, communication is not an optional component of treatment but a core ethical intervention that shapes understanding, trust, adherence, and engagement. Similarly, patient-reported outcomes can be ethically valuable tools when used to support shared understanding rather than translating the patient’s experience into numerical scores. Nutritional care is embedded within supported self-management and shared decision-making, because diet-related choices are shaped by symptoms, patient values, cultural context, resources, access to dietetic support, and the risks of both undertreatment and excessive restriction.

In IBS, self-management is not only a practical necessity, but also an ethically complex aspect of everyday decision-making. Patients frequently make therapeutic choices in the context of uncertainty, fluctuating symptoms, and social influences. These conditions can generate value conflicts. A patient-centered ethical framework should therefore aim to support relational autonomy without shifting responsibility onto patients.

Ultimately, integrating ethics into routine IBS care requires consistent, practice-level commitments such as respectful and structured communication, supported and not mandated self-management, individualized nutritional counseling, and equitable access to trustworthy, comprehensible resources. Sustained therapeutic alliance is particularly important in IBS, where uncertainty, stigma, and food-related fears can undermine confidence and trust. Together, these commitments can reduce unnecessary suffering, strengthen engagement, preserve dietary diversity, and align management with the core aims of patient-centered care.

Future research should aim to empirically evaluate the proposed ethical framework in routine IBS care. This could include qualitative studies exploring patient and clinician perspectives, interventional studies assessing communication-based or PRO-informed care pathways, and implementation studies examining feasibility in gastroenterology clinics. The framework may also inform educational programs for healthcare professionals by supporting training in validation, non-stigmatizing explanations of the gut–brain axis, shared decision-making, nutrition-sensitive communication, and supported self-management. In this way, the ethical framework proposed in this review may serve not only as a conceptual model, but also as a basis for future research, clinical implementation, and professional training in patient-centered IBS care.

## Figures and Tables

**Table 1:** Principal patient-reported outcome instruments used in irritable bowel syndrome care.

Instrument	Main Domains	Strengths	Limitations
Adequate Relief measure [60]	Global symptom relief	Very brief; easy to use	Does not assess specific symptoms or quality of life
IBS Severity Scoring System (IBS-SSS) [61]	Abdominal pain severity/frequency, bloating/distension, bowel dissatisfaction, life interference	Widely used; useful for symptom severity and treatment response	Limited detail on psychosocial burden
IBS Quality of Life questionnaire (IBS-QOL) [65,66]	Dysphoria, interference with activity, body image, health worry, food avoidance, social reaction, sexual concerns, relationships	IBS-specific; captures broader quality-of-life impact	Longer and less practical in busy clinics

## Data Availability

This article is a review and does not report any new data.
